# Blood Cultures for the Diagnosis of Infective Endocarditis: What Is the Benefit of Prolonged Incubation?

**DOI:** 10.3390/jcm10245824

**Published:** 2021-12-13

**Authors:** Vincent Fihman, Hélène Faury, Amina Moussafeur, Raphaelle Huguet, Adrien Galy, Sébastien Gallien, Pascal Lim, Raphaël Lepeule, Paul-Louis Woerther

**Affiliations:** 1 Bacteriology and Infection Control Unit, Department of Prevention, Diagnosis, and Treatment of Infections, Henri-Mondor University Hospital, AP-HP, 94000 Creteil, France; helene.faury@aphp.fr (H.F.); paul-louis.woerther@aphp.fr (P.-L.W.); 2 EA 7380 Dynamyc, EnvA, UPEC, Paris-Est University, 94000 Creteil, France; 3 Department of Cardiovascular Medicine, Henri-Mondor University Hospital, AP-HP, 94000 Creteil, France; amina.moussafeur@yahoo.fr (A.M.); raphaelle.huguet@aphp.fr (R.H.); pascal.lim@aphp.fr (P.L.); 4 SOS Endocardites Unit, Henri-Mondor University Hospital, AP-HP, 94000 Creteil, France; adrien.galy@aphp.fr (A.G.); sebastien.gallien@aphp.fr (S.G.); raphael.lepeule@aphp.fr (R.L.); 5 Antimicrobial Stewardship Team, Department of Prevention, Diagnosis, and Treatment of Infections, Henri-Mondor University Hospital, AP-HP, 94000 Creteil, France; 6 Department of Infectious Diseases, Henri-Mondor University Hospital, AP-HP, 94000 Creteil, France

**Keywords:** infective endocarditis, time-to-positivity blood culture, prolonged incubation, *Cutibacterium acnes*

## Abstract

To assess the need for prolonged incubation of blood culture bottles beyond five days for the diagnosis of infectious endocarditis (IE), we conducted a retrospective cohort study of 6109 sets of two blood culture bottles involving 1211 patients admitted to the Henri Mondor University Hospital for suspicion of IE between 1 January 2016 and 31 December 2019. Among the 322 patients with IE, 194 had positive blood cultures in our centre. Only one patient with a time-to-positivity blood culture of more than 120 h (5 days) was found. The main cause for the 22 patients with positive blood cultures after five days was contamination with *Cutibacterium acnes*. Our results do not support extending the duration of incubation of blood culture bottles beyond five days for the diagnosis of infectious endocarditis, with the exception of patients with risk factors for *C. acnes* infection.

## 1. Introduction

Evidence of sustained bacteraemia is the cornerstone of the diagnosis of infective endocarditis (IE). However, the most recent international guidelines for the diagnosis of this disease do not provide clear instructions on the duration of incubation of blood culture bottles following collection [1,2]. As a result, it has been suggested that the detection of fastidious Gram-negative bacilli of the HACEK group or *Brucella* spp. may require prolonged incubation [2]. A previous review on IE due to rare and fastidious bacteria reported that an incubation of 8 days is required to detect IE due to *Aggregatibacter actinomycetemcomitans* and 21 days for IE due to *Brucella* spp. [3]. The last version of the European recommendations for clinical microbiology laboratories advised a duration of incubation of 7 to 15 days [4], whereas a multicentre study showed that incubation beyond five days was unnecessary for the bacterial documentation of endocarditis due to HACEK [5].

We conducted a retrospective cohort study to evaluate the impact of increasing the duration of incubation of blood cultures to 15 days versus the standard five-day protocol for the bacterial documentation of IE.

## 2. Materials and Methods

The Henri-Mondor University Hospital of Créteil is a reference centre for IE for a population of 1.7 million inhabitants. It includes a team that has been devoted to the treatment of IE since December 2015 [6]. We retrospectively analysed the local IE registry from 1 January 2016 to 31 December 2019 to review demographic information, bacterial or fungal documentation, localisation of vegetation, delays of diagnosis, and antimicrobial treatment. We also reviewed the clinical microbiology laboratory records for all cases of delayed blood culture positivity beyond five days of incubation over the same study period. This study was approved by our local ethics committee.

The diagnosis of IE was made according to the European Society of Cardiology 2015 algorithm, which considered the modified Duke criteria and additional imaging criteria to allow classification of cases with high suspicion [1]. Both definite and possible IE were retained in our study as IE cases. For patients with suspected endocarditis, the minimum diagnostic kit at our institution included three pairs of blood culture vials (Bact’ALERT, bioMérieux, Marcy l’Étoile, France) incubated aerobically and anaerobically, and systematic serological testing for *Coxiella burnetii*, *Bartonella* spp., *Brucella* spp., and *Legionella pneumophila*. In our institution, blood culture vials were incubated for five days from the time they were received by the laboratory. In case of suspected IE based on clinical and imaging criteria [1], the IE team informed the laboratory of the need to prolong the incubation of the patient’s blood culture bottles for a total of 15 days.

In patients undergoing cardiac surgery, excised cardiac valvular tissues were sent for microbiological and pathological analysis. Histological criteria for endocarditis combined inflammatory and destructive tissue lesions with evidenced microorganism colonies on cultures. Culture of tissue biopsies was optimized by using the bead mill processing preanalytical method (Ultra-Turrax^®^; IKA^®^-Werke GmbH & Co. KG, Staufen, Germany), several solid agar plates inoculated for aerobic and anaerobic culture and two blood culture vials per sample (bioMérieux, Marcy l’Étoile, France). Pathogen identification was performed using matrix assisted laser desorption/ionization-time of flight (MALDI-TOF) mass spectrometry (Microflex; Bruker Daltonics, Bremen, Germany). Broad-spectrum 16S ribonucleic acid gene real-time polymerase chain reaction and sequence analysis were performed on surgical samples negative in culture for all patients with suspicion of IE. To that end, we used the UMD-Universal kit according to the instructions of the manufacturer (Molzym GmbH & Co. KG, Bremen, Germany).

Categorical variables are presented as counts (%). Times to positivity of the blood culture are presented as median (min-max) or median (interquartile range, IQR). The differences between groups were assessed with the Mann-Whitney U test for non-parametric independent samples. A *p*-value smaller than 0.05 was considered statistically significant. Statistical analyses were performed using the kGraphPad Prism 5.02 software (GraphPad Software, Inc., La Jolla, CA, USA).

## 3. Results

### 3.1. Description of the Cohort

Over the study period, 140,629 sets of two blood culture bottles for aerobic and anaerobic incubation were analysed by our clinical microbiology laboratory. Among them, the incubation was prolonged for 6109 sets involving 1211 patients with a clinical suspicion of IE, as defined by international guidelines [1].

During the study period, 322 IE cases from 1211 suspected cases were diagnosed according to the current recommendations [1]. Of the 1211 patients suspected of endocarditis, 246 (20.3%) underwent cardiac surgery, of which 78 were finally diagnosed as suffering from IE. Meanwhile, of the 322 IE cases, 185 (57%) underwent cardiac surgery. We identified 254 cases of definite IE and 68 cases of possible IE. Eighty-seven IE cases (27%) were on prosthetic heart valves, of which 59 occurred on bioprosthetic valves, 23 on mechanical prostheses, and five after mitral annular reconstruction. At the time of IE diagnosis, 88 patients (27%) had an intracardiac device: 65 (20%) had a pacemaker and 23 (7%) had an implantable cardioverter defibrillator.

### 3.2. Microbiological Results

The average number of bottles analysed per patient with suspected IE was >10 (i.e., >5 pairs per patient). Among the 322 IE cases, 128 patients had negative blood cultures in our institution. Of these, 104 had been on antibiotics at admission for a median of six days (min-max: 0–110 days). Instead, the causative microorganism identification was obtained by positive blood cultures in a primary care centre for 62 patients (48%), heart-valve culture for 31 (24%), molecular diagnosis on vegetation or valves for 17 (13%), and serology for four (3%).

No microorganism was identified for 14 patients (11%). Of these 14 patients, all had been investigated by the standard kit for the diagnosis of IE and eight had undergone surgery but the samples were negative in both culture and RT-PCR despite intraoperative and histological findings in favour of IE. Of the 14 patients, eight had received antibiotic therapy prior to admission into our hospital. In all cases, the IE treatment was continued to completion.

Thus, 194 cases of IE were identified by positive blood cultures in our clinical microbiology laboratory. Table 1 shows the epidemiology of the microorganisms found in the blood cultures and their time to positivity by bacterial group.

### 3.3. Influence of Antimicrobial Treatment

At the time the blood cultures were drawn, 103 of 194 patients were already taking antibiotics that were active against the microorganism subsequently identified in the blood cultures for a median of one day (min-max: 0–21 days). This could affect the time to positivity, even if the bottles contain resins that inhibit the antibiotics [7]. Active antibiotic treatment did not change the time to positivity of the aerobic blood culture bottles. Indeed, the median time to positivity with or without antibiotics was 15.3 h, (IQR 10.7–20.0) vs. 14.4 h, (IQR 11.5–20.2), respectively (p = 0.9848 by the two-tailed Mann Whitney U test). In contrast, the time to positivity of the anaerobic bottles was longer if an active antibiotic was being taken, with a median time to positivity with or without antibiotics of 20.6 h, (IQR 14.6–30.2) vs. 15.7 h (IQR 12.7–24.2), respectively (p = 0.0114 by the two-tailed Mann Whitney U test).

### 3.4. Influence of Extended Incubation of Blood Culture Vials

Extended incubation allowed the identification of 32 bacteria between 5 and 15 days of incubation for 29 patients from 1211 suspected IE cases. No fungi were detected after five days (Supplementary Table S1).

For seven patients, three or more other blood culture bottles had a time to positivity of <5 days for the same microorganism. In the 22 remaining patients, a single bottle among all sampled showed the presence of *Cutibacterium acnes* (*n* = 18, 82%), *Actinomyces naeslundi* (*n* = 2, 9%), *Micrococcus* spp. (*n* = 1), or *Staphylococcus pettenkoferi* (*n* = 1). Analysis of the medical records of the 22 patients showed that the diagnosis of IE had been ruled out in 14 patients (64%). Another microorganism was thought to be the cause of IE in seven patients (32%) due to sustained bacteraemia with the other species, associated in two cases with a positive culture of intraoperative heart valve biopsies. In only one case (patient no. 23, Supplementary Table S1), an anaerobic bottle positive with *C. acnes* after 275.3 h of incubation was consistent with mediastinitis and definite prosthetic aortic valve endocarditis associated, later confirmed by *rrs* sequencing on intraoperative specimens.

## 4. Discussion

To the best of our knowledge, we present here the first analysis of time to positivity blood culture from a large monocentric cohort of more than 1211 patients with a clinical suspicion of IE. We have shown that, among the 322 cases of IE, 194 were identified in our laboratory by positive blood culture and the time to positivity was less than 5 days in all but seven cases. For six out of these seven cases, the diagnosis had already been made from other vials that had previously tested positive for the same bacteria, but with a time to positivity of less than 5 days (Supplementary Table S1). In only one case of *C. acnes* infection was the prolonged incubation period useful for diagnosis. In addition, active antibiotic treatment started recently at the time of blood culture collection did not significantly increase the time to positivity of aerobic bottles. Even though this antibiotic treatment significantly increased the time to positivity of the anaerobic bottles, it was still less than 5 days.

A recent study suggested that prolonged incubation of blood culture bottles improves the diagnosis of endocarditis due to *C. acnes* on prosthetic valves or cardiac implantable device in male patients [8]. In our experience, prolonged incubation of the blood culture bottles to 15 days (360 h) allowed for documenting, in only one case, a definite prosthetic aortic valve endocarditis but increased contamination rate due to this bacterial species, as described for cultures of bone and joint specimens [9]. Furthermore, this prolonged incubation did not increase the detection of bacteria responsible for IE, including those due to microorganisms generally considered as difficult to culture, such as nutritionally variant streptococci, HACEK group bacteria, or Candida spp. Regarding the HACEK group, our study provided some evidence that prolonged incubation of 6109 blood culture bottle sets did not result in the diagnosis of new cases of IE caused by these bacteria, in accordance with a previous multicentre study conducted on 407 sets [5].

Our study had several limitations. First, we did identify only one *Brucella* spp., as our centre is in a region with a low incidence of Brucellosis. Although *Brucella* spp. may require prolonged incubation of blood culture bottles, published data appear to be contradictory [10]. Second, the study was conducted using BacT/Alert (bioMérieux, Marcy l’Etoile, France) blood culture bottles, which were loaded into a BacT/Alert 3D^®^ system as soon as they were received. The time to positive detection could be different for other automated systems, such as the VIRTUO^®^ system (bioMérieux) [11], or with Bactec™ (Becton Dickinson Instrument Systems, Sparks, MD, USA) blood-culture bottles [12], making our results difficult to extrapolate. Indeed, published data appear to show shorter times to positivity with these more recent systems. However, the hypothesis that there is no need to prolong the incubation of blood culture bottles would be strengthened. Finally, because our study was monocentric, it may be difficult to generalize our results. However, this approach allowed us to control technical biases and the local epidemiology of IE we diagnosed was equivalent to that found in a recent French multicentre study [13].

Our study provides data arguing against a broad recommendation to extend the duration of incubation of blood culture bottles for the diagnosis of IE. This procedure should be reserved for patients with risk factors for endocarditis due to *C. acnes*, i.e., male patients with a prosthetic valve or intra-cardiac device. These results should be considered in establishing a quality assurance system for blood cultures that satisfies the requirements of accreditation and focuses on patient needs [14]. Progress in the diagnosis of IE pathogens relies on a bundled approach that includes optimisation of pre-analytical parameters (e.g., volume of vial filling, number of vials collected, sampling prior to antibiotic injection, skin preparation), rapid start of incubation, reorganisation (e.g., 24/7 transport service) [15] and close involvement with the IE team to identify patients for whom extended incubations would be necessary. In addition, our results could help to prevent the overfilling of automated blood culture incubators and the subsequent risk of missing the detection of clinically significant bacteraemia [16].

## Figures and Tables

**Table 1:** Distribution of causative microorganisms of patients with infective endocarditis and blood culture time to positivity.

Microorganisms	No (%) of Patients (*n* = 194)	Time to Positivity of the First Aerobic Blood Culture Bottle (Median (Min–Max) in Hours)	Time to Positivity of the First Anaerobic Blood Culture Bottle (Median (Min–Max) in Hours)
*S. aureus*	68 (35.1)	13.4 (6.4–38.7)	18.95 (9.6–55.4)
Coagulase Negative staphylococci ^1^	14 (7.2)	20.7 (15.3–44.0)	31.3 (17.5–144.7)
*Enterococcus* spp. ^2^	41 (21.1)	13.4 (3.6–56.4)	14.8 (4.1–103.3)
*S. mitis* group	21 (10.8)	15.2 (8.7–45.6)	17.8 (9.7–80.2)
*S. bovis* group ^3^	15 (7.7)	16.6 (12.2–51.3)	14.1 (8.6–38.2)
beta-haemolytic streptococci (A, B, C, G) ^4^	5 (2.6)	7.7 (6.8–9.5)	9.9 (7.9–21.0)
*S. milleri* group ^5^	5 (2.6)	22.9 (13.0–26.1)	34.7 (16.5–62.7)
Nutritionally variant streptococci ^6^	4 (2.1)	20.6 (17.9–32.4)	31.8 (23.4–38.4)
*S. salivarius* group	2 (1.0)	20.5 (16.8–24.2)	22.9 (18.2–27.7)
Other *S. viridans* ^7^	1 (0.5)	13.9	16.4
*Corynebacterium jeikeium*	1 (0.5)	47.8	No data
Enterobacterales ^8^	3 (1.5)	14.6 (12.1–17.1)	11.0 (9.3–12.7)
HACEK group ^9^	1 (0.5)	75.0	96.8
Other bacteria ^10^	6 (3.1)	38.75 (13.1–63.1)	19.7 (13.6–275.3)
Polymicrobial ^11^	5 (2.6)	10.7 (8.3–41.9)	11.9 (8.8–47.2)
Yeast/fungi ^12^	2 (1.0)	71.9 (35–108.9)	No data

^1^*S. epidermidis* (*n* = 8), *S. lugdunensi*s (*n* = 3), *S. capitis* (*n* = 1), *S. xylosus* (*n* = 1), *S. warneri* (*n* = 1); ^2^ *E. faecalis* (*n* = 39) and *E. faecium* (*n* = 2); ^3^ *S. gallolyticus* (*n* = 12) and *S. infantarius* (*n* = 3); ^4^ *S. pyogenes* (*n* = 1), *S. agalactiae* (*n* = 3) and *Streptococcus dysgalactiae* subsp. Equisimilis (*n* = 1); ^5^ *S. anginosus* (*n* = 3), *S. constellatus* (*n* = 1) and *S. intermedius* (*n* = 1); ^6^ *Aerococcus urinae*, *Granulicatella adiacens*, *Granulicatella elegans*, and *Abiotrophia defective* (1 strain of each species); ^7^ *S. suis* (*n* = 1); ^8^ *E. coli* (*n* = 2) and *K. pneumoniae* (*n* = 1); ^9^ *Cardiobacterium hominis* (*n* = 1); ^10^ *Bacillus cereus*, *Brucella melitensis*, *Clostridium perfringens*, *Cutibacterium acnes*, *Lactobacillus rhamnosus*, and *Lactococcus garviae* (1 strain of each species); ^11^ *S. aureus* plus *S. agalactiae* (*n* = 1), *E. coli* plus at least one other pathogen (*n* = 4); ^12^ *C. albicans* (*n* = 1) and *C. parapsilosis* (*n* = 1).

## Data Availability

The data presented in this study are available upon request from the corresponding author. The data are not publicly available because of the confidentiality of health data, in accordance with the French recommendations of the Commission Nationale Informatique et Libertés (CNIL).

## Supplementary Materials

The following are available online at https://www.mdpi.com/article/10.3390/jcm10245824/s1, Table S1: Description of 29 patients who had positive blood cultures beyond 5 days of incubation.
