# Supplementary material for: Blood Cultures for the Diagnosis of Infective Endocarditis: What Is the Benefit of Prolonged Incubation?
Source: J Clin Med. 2021 Dec 13;10(24):5824. doi: 10.3390/jcm10245824 (PMC8705825; doi:10.3390/jcm10245824)
Supplement: Supplementary file 1 [file jcm-10-05824-s001.zip › jcm-1471313-supplementary.pdf]

**Table S1.** Description of 29 patients who had positive blood cultures beyond 5 days of incubation.

| Patient no. | Sex, Age (in years) | Bacteria Identified | TTP [1] | Results of other Blood Cultures or Positive Serology | Cardiac Surgery Performed | Results on Cardiac Intraoperative Samples | Cardiac Imaging Findings | Minor Criteria for IE [2] Diagnosis | Other Findings | Diagnosis Retained |
|---|---|---|---|---|---|---|---|---|---|---|
| **1** | M, 73 | Abiotrophia defectiva | 161.1 | A further 17 positive vials with the same species had delays of <120 hours. | Yes | A. defectiva positive culture | TOE [3]: aortic ring abscess | Fever > 38°C | None | Definite IE due to A. defectiva |
| **2** | M, 58 | Aerococcus urinae | 164.9 | A further 3 positive vials with the same species had delays of <120 hours. | No | NA | TOE: vegetation (15 mm) on native mitral valve | Fever > 38°C | None | Definite IE due to A. urinae |
| **3** | F, 81 | Enterococcus faecalis | 254.6 | A further 16 positive vials with the same species had delays of <120 hours. | Yes | E. faecalis positive culture | TOE: Vegetation (9 mm) on transcatheter aortic valve | Fever > 38°C | None | Definite IE due to E. faecalis |
| **4** | F, 71 | Granulicatella elegans | 159.9 | A further 7 positive vials with the same species had delays of <120 hours. | Yes | G. elegans positive culture | TOE: vegetation (3 mm) on native aortic valve | Fever > 38°C | None | Definite IE due to G. elegans |
| **5** | F, 89 | Staphylococcus epidermidis | 144.7 | A further 6 positive vials with the same species had delays of <120 hours. | No | NA | TOE: vegetation on pacemaker leads | Fever > 38°C | None | Definite IE due to S. epidermidis |
| **6** | F, 55 | Streptococcus dysgalactiae spp. equisimilis | 168.5 | A further 4 positive vials with the same species had delays of <120 hours in a primary care hospital | No | NA | None | Fever > 38°C | Diabetic foot osteitis on X-ray and positive bone biopsy culture with S. dysgalactiae spp. equisimilis | **Rejected** IE. Diabetic foot osteitis. |
| **7** | | Streptococcus sanguinis | 170.7 | A further 4 positive vials with the same species had delays of <120 hours. | Yes | S. sanguinis positive culture | TOE: aortic ring abscess | Fever > 38°C | None | Definite IE due to S. sanguinis |

| | | | | | | | | | | |
|---|---|---|---|---|---|---|---|---|---|---|
| **8** | F, 58 | Actinomyces naeslundii | 253.0 | A further 5 negative vials | No | NA | TTE: No abnormalities in two 7-day separate examinations. | None | Presence of a heart murmur. Recovery without antibiotic therapy. | **Rejected** IE |
| **9** | M, 56 | Actinomyces naeslundii | 332.4 | A further 7 negative vials and two positive vials with Staphylococcus aureus in a primary care hospital | Yes | S. aureus positive culture | TTE: vegetation (18 mm) on native aortic valve | Fever > 38°C, splenic emboli | None | Definite IE due to S. aureus |
| **10** | M, 66 | Cutibacterium acnes | 142.7 | A further 7 negative vials and two positive vials with Staphylococcus aureus in a primary care hospital | Yes | S. aureus positive culture | TTE: intracardial abscess and fistula | Fever > 38°C | Bacteraemia related to subcutaneous implanted port catheter as evidenced by positive catheter culture with S. aureus | Definite IE due to S. aureus |
| **11** | M, 57 | Cutibacterium acnes | 174.8 | A further 3 negative vials and two positive vials with Bacteroides fragilis in 28,2 and 28,7 hours, respectively | No | NA | TTE: No abnormalities in two 7-day separate examinations. | None | Multiorgan failure in a patient with sickle cell disease | Rejected IE |
| **12** | M, 64 | Cutibacterium acnes | 190.4 | A further 13 negative vials | No | NA | TOE: fortuitous discovery of a vibratile image on the aortic valve during a heart insufficiency evaluation. | None | None | Rejected IE. Suspicion of cardiac papillary fibroelastoma. |
| **13** | M, 44 | Cutibacterium acnes | 205.0 | A further 10 negative vials and one positive vial with Corynebacterium jeikeium in 47,8 hours | Yes | C. jeikeium positive culture | TOE: vegetation (6 mm) on native mitral valve | None | Recent dental procedures performed with well-conducted antibiotic prophylaxis | Definite IE due to C. jeikeium |

| | | | | | | | | | | |
|---|---|---|---|---|---|---|---|---|---|---|
| | | | | | | | | | with amoxicillin | |
| **14** | M, 72 | Cutibacterium acnes | 213.8 | A further 5 negative vials | No | NA | TTE: No abnormalities in two 7-day separate examinations. | Fever > 38°C | Recovery without antibiotic therapy. No relapses after one year of follow-up | **Rejected** IE |
| **15** | M, 48 | Cutibacterium acnes | 219.5 | A further 15 negative vials and one positive vial with Staphylococcus hominis spp. hominis in 21,0 hours | No | NA | TOE: vibratile image on the mitral valve | Intracranial haemorrhage | Recovery without antibiotic therapy. No relapses after two years of follow-up | **Rejected** IE. Suspicion of cardiac papillary fibroelastoma. |
| **16** | M, 39 | Cutibacterium acnes | 230.3 | A further 5 negative vials | No | NA | TTE: No abnormalities in two 7-day separate examinations in a patient with a biological mitral prosthesis | Fever > 38°C | Recovery without antibiotic therapy. No relapses after two years of follow-up | **Rejected** IE |
| **17** | M, 83 | Cutibacterium acnes | 245.1 | A further 4 positive vials with Staphylococcus lugdunensis in a primary care hospital | Yes | S. lugdunensis positive culture | TOE: vegetation on pacemaker leads | Fever > 38°C | Simultaneous prosthetic hip infection due to S. lugdunensis | Definite IE due to S. lugdunensis |
| **18** | F, 63 | Cutibacterium acnes | 245.5 | A further 13 negative vials | No | NA | TTE: No abnormalities in two 7-day separate examinations. | Fever > 38°C | Erysipelas. Presence of a heart murmur. | **Rejected** IE |
| **19** | M, 71 | Cutibacterium acnes | 254.9 | A further 15 positive vials with Streptococcus gallolyticus | Yes | S. gallolyticus positive culture | TOE: vegetation (10 mm) on native aortic valve | Fever > 38°C, Intracranial infectious (mycotic) aneurysm | None | Definite IE due to S. gallolyticus |

| | | | | | | | | | | |
|---|---|---|---|---|---|---|---|---|---|---|
| **20** | M, 88 | Cutibacterium acnes | 263.0 | A further 4 positive vials with Staphylococcus aureus from 2 separate blood cultures | No | NA | TTE: No abnormalities in two 7-day separate examinations. | Fever > 38°C | Bilateral parotiditis. Recovery and no relapse of bacteraemia two months after stopping a two-week course of cefazolin | **Rejected** IE |
| **21** | M, 73 | Cutibacterium acnes | 271.5 | A further 7 negative vials and four positive vials with Staphylococcus aureus in a primary care hospital | No | NA | TTE: No abnormalities in two 7-day separate examinations. | Fever > 38°C | Diabetic foot osteitis on X-ray | **Rejected** IE |
| **22** | M, 43 | Cutibacterium acnes | 272.0 | A further 9 positive vials with Streptococcus mitis group | Yes | S. mitis group positive culture | TOE: vegetation (15 mm) on native mitral valve | Fever > 38°C, intracranial and splenic emboli, Osler's nodes | Presence of a heart murmur. | Definite IE due to S. mitis group |
| **23** | M, 62 | Cutibacterium acnes | 275.3 | A further 5 negative vials | Yes | C. acnes positive 16S RT-PCR | Abnormal activity around the site of prosthetic valve implantation detected by 18F-FDG PET/CT (Bentall procedure was performed for >1 year) | Intracranial haemorrhage | None | Definite IE due to C. acnes |
| **24** | M, 30 | Cutibacterium acnes | 284.0 | A further 7 positive vials with Staphylococcus aureus | No | NA | TTE: No abnormalities in two 7-day separate examinations. | Fever > 38°C | Bacteraemia related to femoral dialysis catheter infection as evidenced by positive catheter culture | **Rejected** IE |

| | | | | | | | | | | |
|---|---|---|---|---|---|---|---|---|---|---|
| **25** | M, 61 | Cutibacterium acnes | 317.3 | A further 13 negative vials | No | NA | TTE: No abnormalities in two 7-day separate examinations. | Intracranial haemorrhage | None | **Rejected** IE |
| **26** | F, 54 | Cutibacterium acnes | 318.7 | A further 9 negative vials | Yes | Culture and 16S RT-PCR negative | TTE: No abnormalities in two 7-day separate examinations in a patient with a mechanical mitral prosthesis | Fever > 38°C | Recovery without antibiotic therapy. No relapses after two years of follow-up | **Rejected** IE |
| **27** | M, 84 | Cutibacterium acnes | 361.3 | A further 7 negative vials | No | NA | TTE: No abnormalities in two 7-day separate examinations. | Fever > 38°C | Presence of a heart murmur. Recovery without antibiotic therapy. | **Rejected** IE |
| **28** | F, 39 | Micrococcus sp. | 166.3 | A further 8 positive vials with Staphylococcus aureus in a primary care hospital | No | NA | TTE: vegetation (17 mm) on native pulmonary valve | Fever > 38°C | None | Definite IE due to S. aureus |
| **29** | F, 65 | Staphylococcus pettenkoferi | 205.0 | A further 5 negative vials | No | NA | TTE: No abnormalities in two 7-day separate examinations. | Fever > 38°C | Recovery without antibiotic therapy. No relapses after four years of follow-up | **Rejected** IE |

**[1] TTP: Time to positivity of blood culture bottle (in hours);**

**[2] IE: infective endocarditis ;**

**[3] TOE: transoesophageal echocardiography**
